# Pomegranate (*Punica granatum L*.) peel extract ameliorates metabolic syndrome risk factors in patients with non-alcoholic fatty liver disease: a randomized double-blind clinical trial

**DOI:** 10.1186/s12937-023-00869-2

**Published:** 2023-08-22

**Authors:** Hanieh Barghchi, Narges Milkarizi, Saba Belyani, Andisheh Norouzian Ostad, Vahid Reza Askari, Farnood Rajabzadeh, Ladan Goshayeshi, Seyede Yegane Ghelichi Kheyrabadi, Maryam Razavidarmian, Zahra Dehnavi, Seyyed Reza Sobhani, Mohsen Nematy

**Affiliations:** 1 Department of Nutrition, Faculty of Medicine, Mashhad University of Medical Sciences, Mashhad, Iran; 2 Student Research Committee, Mashhad University of Medical Sciences, Mashhad, Iran; 3 Metabolic Syndrome Research Center, Department of Nutrition, School of Medicine, Mashhad University of Medical Sciences, Mashhad, Iran; 4 Student Research Committee, North Khorasan University of Medical Sciences, Bojnourd, Iran; 5 Pharmacological Research Center of Medicinal Plants, Mashhad University of Medical Sciences, Mashhad, Iran; 6 Neurogenic Inflammation Research Centre, Mashhad University of Medical Sciences, Mashhad, Iran; 7 Department of Radiology, Mashhad Medical Sciences Branch, Islamic Azad University, Mashhad, Iran; 8 Department of Gastroenterology and Hepatology, Faculty of Medicine, Mashhad University of Medical Sciences, Mashhad, Iran; 9 Gastroenterology and Hepatology Research Center, Mashhad University of Medical Sciences, Mashhad, Iran; 10 Department of Nutrition Sciences, Varastegan Institute for Medical Sciences, Mashhad, Iran

**Keywords:** Pomegranate Peel, Metabolic syndrome, Fatty liver, Dyslipidemia, Hypertension

## Abstract

**Introduction:**

Non-alcoholic fatty liver disease (NAFLD) is a metabolic syndrome (MS)-related liver disorder that has an increasing prevalence. Thus, the aim of our study is to evaluate the effects of pomegranate peel extract (PP) supplementation on hepatic status and metabolic syndrome risk factors.

**Methods:**

In phase one, the hydro-alcoholic extraction of the peel of 750 kg of pomegranate (*Punica granatum L*.) was performed by the soaking method. Then, in phase two, NAFLD patients received 1500 mg of placebo (n = 37) or pomegranate peel capsules (n = 39) with a 500-kcal deficit diet for 8 weeks. Gastrointestinal intolerance, dietary intake, lipid and glycemic profiles, systolic and diastolic blood pressure, body composition, insulin resistance indexes, and elastography-evaluated NAFLD changes were followed.

**Results:**

The mean age of participants was 43.1 ± 8.6 years (51.3% female). Following the intervention, the mean body weight (mean changes: -5.10 ± 2.30 kg), waist circumference (-7.57 ± 2.97 cm), body mass index (-1.82 ± 0.85 kg/m^2^), body fat index (-1.49 ± 0.86), and trunk fat (− 3.93 ± 3.07%), systolic (-0.63 ± 0.29 cmHg) and diastolic (-0.39 ± 0.19 cmHg) blood pressure, total cholesterol (-10.51 ± 0.77 mg/dl), triglyceride (-16.02 ± 1.7 mg/dl), low-density lipoprotein cholesterol (-9.33 ± 6.66 mg/dl; all P < 0.001), fat free mass (− 0.92 ± 0.90 kg; P < 0.003), and fasting blood sugar (-5.28 ± 1.36 mg/dl; P = 0.02) decreased significantly in PP in contrast to the placebo group in the raw model and when adjusted for confounders. Also, high-density lipoprotein cholesterol (5.10 ± 0.36 mg/dl), liver steatosis and stiffness (− 0.30 ± 0.17 and − 0.72 ± 0.35 kPa, respectively, all P < 0.001) improved in the PP group. However, fasting insulin (P = 0.81) and homeostatic model assessment for insulin resistance (HOMA-IR) (P = 0.93) were not significantly different when comparing two groups during the study in the raw and even adjusted models.

**Conclusion:**

In conclusion, 1500 mg pomegranate peel extract along with a weight-loss diet improved metabolic syndrome risk factors and reduced hepatic steatosis in patients with NAFLD after 8 weeks.

## Introduction

Non-alcoholic fatty liver disease (NAFLD) is a spectrum of liver disease ranging from isolated steatosis to steatohepatitis (NASH), which can eventually progress to NASH-fibrosis, cirrhosis and hepatocellular carcinoma [1]. It is also known as a multi-system disease affecting various extra-hepatic organs, subsequently leading to high morbidity and mortality, with global prevalence estimated between 25 and 38% [1, 2]. The detailed pathophysiology of NAFLD is complicated and involves heterogeneous exogenous and endogenous factors, which involved lifestyle, nutritional factors, lipogenesis, cell death, chronic low-grade inflammatory response and an altered gut microbiome [3, 4]. NAFLD is an obesity-related metabolic liver disorder considered a hepatic element of metabolic syndrome (MS) and has been associated with obesity, insulin resistance, type 2 diabetes, dyslipidemia, hypertension, and cardiovascular disease [5]. Currently, there are no approved drugs to specifically target or treat NAFLD. Weight loss, achieved through change in dietary and physical activity behaviors is the recommended treatment for NAFLD, which can reduce liver fat, inflammation and fibrosis [6, 7]. The Mediterranean diet has been effective in reducing metabolic syndrome and CVD risk factors, as well as NAFLD improvement by reduction of the severity of hepatic steatosis [8–10]. A plant-based diet, with significant fiber, anti-oxidants, vegetable proteins, polyunsaturated, and monounsaturated fat have been emphasized [11].

Several studies in recent years have revealed that dietary components or medicinal plants (with pharmacological capabilities) could be thought of as a substitute for traditional NAFLD management methods [12–16].

Pomegranate fruit (*Punica granatum L*.) has a high level of various phytochemicals such as polyphenols, fatty acids, amino acids, tocopherols, sterols, terpenoids, and alkaloids, due to their therapeutic effects such as anti-inflammatory, antioxidant, and antineoplastic, reducing blood glucose, fat-lowering, anti-hypertension and antimicrobial effects in animal and clinical trials on NAFLD or other diseases [17–23]. The peel of the pomegranate fruit contains the highest amount of phytochemicals in it. About 48 phenolic compounds (flavonoids, hydrolyzed tannins such as ellagitannins and galagyl esters, anthocyanins, gallotannins, hydroxycinnamic acid and hydroxybenzoic acid) have been identified in the skin and other parts of pomegranate [17].

**Fig. 1:**
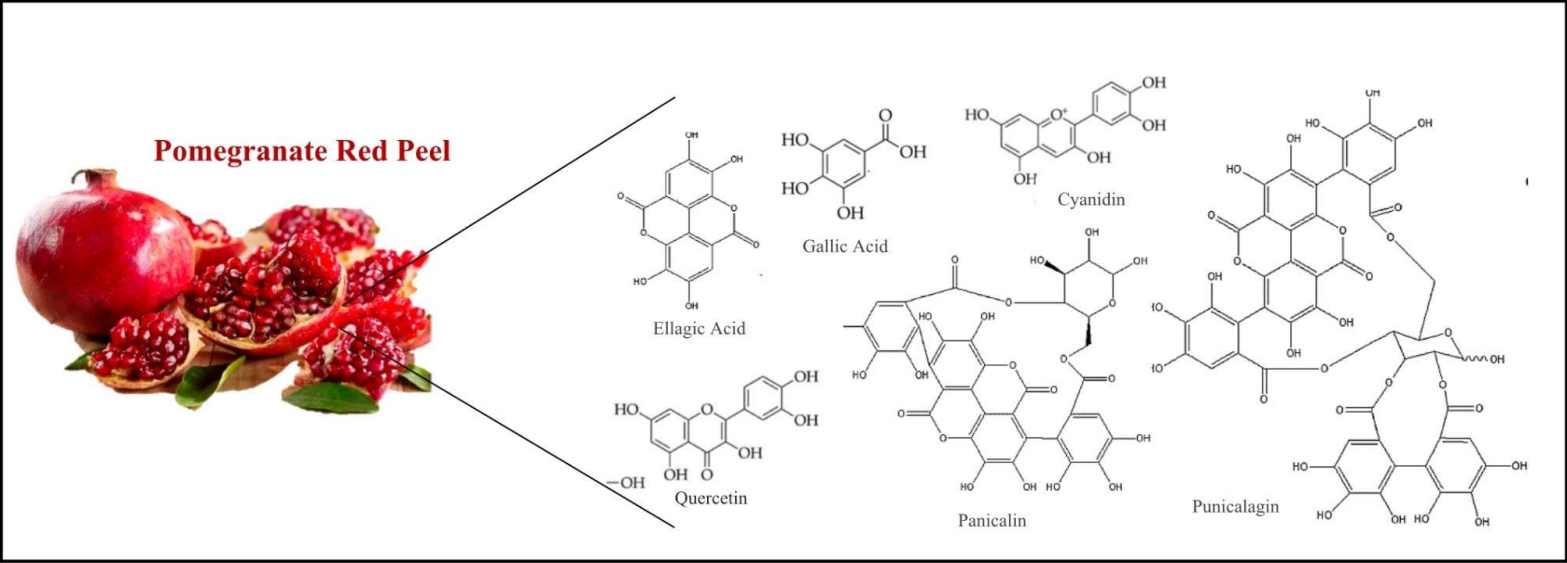
Some of active phenolic contents of Pomegranate Peel

A lot of evidence shows that hydrolysable polyphenols in pomegranate red peel, especially ellagitannins, are the most active antioxidants among the tannins in it, and others such as punicalagin, panicalin and gallic acid have antioxidant and pleiotropic biological activities and especially act synergistically as a strong antioxidant network that causes various beneficial effects [24, 25] (Fig. 1). Also, recent studies show that pomegranate peel, in addition to its antioxidant properties, can be a promising candidate for the treatment of non-alcoholic fatty liver by maintaining the balance of the microbiome of the digestive system and influencing the expression of key genes in the pathways of inflammation and liver lipogenesis and inhibiting the signaling pathways in liver fibrogenesis [26, 27]. Thus, the aim of our study is to evaluate the effects of pomegranate peel extract (PP) supplementation on hepatic status and metabolic syndrome risk factors including lipid profile, blood pressure, insulin and fasting blood glucose, and body composition data such as weight, waist circumferences, body fat percent, trunk fat and lean body mass in patients with non-alcoholic fatty liver disease.

## Materials and methods

### Study design

The present study constitutes a randomized, double-blind, controlled clinical trial aimed to examine the impact of pomegranate peel extract, administered as a dietary supplement over an eight-week duration, on various parameters including inflammatory and oxidative stress markers, nutritional status, and liver fibrosis and steatosis in individuals diagnosed with non-alcoholic fatty liver disease (NAFLD). The study was conducted in Mashhad, Iran, spanning from December 2021 to November 2022, and adhered to the ethical guidelines set forth in the Helsinki Declaration. Approval for the study was obtained from the ethics committee of Mashhad University of Medical Sciences (ethics code: IR.MUMS.MEDICAL.REC.1400.195 and IRCT20210726051988N1).

The study had two phases; first, the extraction of pomegranate peel (PP) hydro-alcoholic extract; second, the randomized clinical trial. The study participants were selected from patients with NAFLD who sought medical attention at the Nutrition Clinic and fulfilled specific inclusion criteria. These criteria encompassed individuals between the ages of 18 and 60 years, diagnosed with liver fibrosis and steatosis through elastography or liver fibro scan, possessing the ability to read and write, and providing informed consent by completing the requisite form. Conversely, individuals were excluded from participation if they were pregnant or lactating, exhibited morbid obesity (with a body mass index exceeding 40), had a history of alcohol consumption surpassing 20 g per day for women or 30 g per day for men, suffered from any immune disorder, including autoimmune disorders, cancer, or human immunodeficiency virus (HIV) infection, experienced liver or kidney failure, presented with other liver diseases such as hepatitis or alcoholic liver disease, utilized hepatotoxic drugs such as sodium valproate, possessed a history of food allergies to pomegranate or herbal supplements, or had undergone bariatric surgery for weight loss.

Throughout the study, participants who voluntarily withdrew from the research, became pregnant, developed hypersensitivity to either the pomegranate peel extract or the placebo, or exhibited less than 70% compliance with their assigned treatment regimen were excluded from further analysis.

At the study’s initiation, all participants provided written informed consent. Demographic and socio-economic information, including education level, family size, housing status, occupation, medical history, and medication and supplement usage, was collected through a questionnaire. The sample size calculation for each group was based on Soleimani et al.‘s research on non-alcoholic fatty liver disease (NAFLD) [28], utilizing a formula for comparing proportions of a qualitative attribute in independent statistical populations. This calculation determined that 39 NAFLD patients were needed per group, considering a significance level (α) of 0.05, study power of 80%, and accounting for a 20% dropout rate [29].

To minimize heterogeneity among the study groups, a blocked randomization design was implemented to allocate participants randomly. The blinding principles were strictly adhered to, ensuring that both the drug and placebo were indistinguishable in terms of color, size, smell, and packaging. This maintained blinding throughout the study and statistical analysis, with only the pharmacist possessing knowledge of the allocation. To conceal the random allocation, coded boxes were utilized. This ensured that neither the participants nor the researchers were aware of the group assignments until the conclusion of the study.

Demographic and socio-economic information about the individuals was obtained using a specific questionnaire at beginning the study. The questionnaire included questions about education level, family size, housing status, occupation, medical history, and use of medications and supplements.

### Fatty liver evaluation

In this study, liver tissue imaging was conducted utilizing the SuperSonic Aixplorer device (SuperSonic Imagine S.A., Aix-en-Provence, France) and the two-dimensional elastography technique. To assess the severity of steatosis, numerical values for the hepatorenal sonographic index were derived from the research conducted by Webb et al. Specifically, the values of 49.1, 86.1, and 23.2 were assigned to mild (grade 1), moderate (grade 2), and severe (grade 3) steatosis, respectively [30].

### Biochemical evaluations

At the beginning and end of the study, 5 cc blood samples were collected from all participants who had fasted for 10–12 h. Serum samples were obtained by centrifuging the blood samples at 3000 rpm for 15 min and immediately stored at -20 °C until testing. Using the Alpha Classic AT Plus analyzer, serum levels of fasting blood glucose, triglycerides, total cholesterol, high-density lipoprotein cholesterol, and low-density lipoprotein cholesterol were evaluated by the colorimetric method. The serum insulin level was measured by the quantitative luminescence method using a diagnostic kit from “Snibe Company” (Shenzhen, China) and the Maglumi 800 analyzer in specialized laboratory. The triglyceride-to-glucose ratio was obtained by logarithmically multiplying the product of triglycerides and half of the fasting plasma glucose, and values greater than or equal to 4.49 were considered indicative of insulin resistance [31]. Additionally, the serum total cholesterol to high-density lipoprotein cholesterol ratio was calculated at the beginning and end of the study.

### Food intake assessment

In this study, dietary intake was assessed using a three-day food record, including two weekdays and one weekend day. To accurately assess the participants’ dietary patterns, they were asked to complete a three-day food record at the beginning of the study and at the end of each month. The energy and nutrient intake of each participant were then calculated using Nutritionist IV software (N-Squared Computing, Salem, OR, USA).

### Anthropometric assessment

In present study, the participants’ height and weight were measured following standardized procedures [32]. Height measurements were obtained using a wall-mounted stadiometer (Seca 206, Germany) with a precision of 0.1 cm, while weight measurements were recorded using a digital floor scale (Seca 813, Germany) with a precision of 0.1 kg. These measurements were taken without shoes and with minimal clothing to ensure accuracy. To assess body composition, the Tanita BC-418 body composition analyzer (Tanita Corporation, Tokyo, Japan) was employed. Additionally, waist circumference was measured with a flexible, non-stretchable tape measure. The measurement was taken at the midpoint between the lower border of the rib cage and the iliac crest while the participants stood with their feet close together and no pressure applied to the body surface. The waist circumference measurement was recorded with an approximate precision of 1.0 cm [32].

### Blood pressure assessment

The blood pressure of the participants was measured at each visit by a non-dominant person using a calibrated and standardized mercury sphygmomanometer (ALPK2) with a standard cuff size of 12 × 25 cm. Blood pressure was also measured using a digital medical device as a secondary measurement method.

### Physical activity assessment

Data on the participants’ physical activity levels were collected using the International Physical Activity Questionnaire-Short form (IPAQ) [33]. The Persian version of this questionnaire has been validated and shown to be reliable [34, 35]. The questionnaire is designed for individuals between the ages of 18 and 65 and encompasses inquiries regarding the duration and intensity of physical activities undertaken over the past seven days. The IPAQ assesses the number of hours engaged in physical activity at different intensities, including walking, moderate-intensity activities, and vigorous-intensity activities. The energy expenditure from physical activities over the course of a week is quantified using Metabolic Equivalent Task (MET) minutes per week. The total MET-minute/week value is obtained by summing the walking, moderate, and vigorous MET-minute/week values. Participants’ physical activity levels were classified as low, moderate, or high based on their total MET-minutes per week. Less than 600 MET-minutes per week were classified as low activity, 600 to 3000 MET-minutes per week as moderate activity, and more than 3000 MET-minutes per week as high activity.

### Gastrointestinal evaluation

Potential gastrointestinal symptoms, were evaluated using the Gastrointestinal Symptom Severity Rating (GSSR) questionnaire [36] at beginning, in the middle and at the end of the study. This questionnaire consists of 15 questions that are scored on a Likert scale ranging from no discomfort (zero) to severe discomfort (seven). It assesses five different dimensions, including abdominal pain (heartburn, hunger pains, nausea), reflux (stomach burning and food regurgitation), diarrhea (loose stools, urgent need to defecate), constipation (difficulty passing stools, painful defecation, sensation of incomplete evacuation), and dyspepsia (abdominal bloating, belching, stomach rumbling, increased abdominal gas) [36].

## Intervention

### The study had 2 phases

#### Pomegranate peel hydro-alcoholic extraction

According to our knowledge, this was the first study in evaluating the effects of PP in NAFLD patients. Thus, we calculate the optimal dose from a related animal study which reported promising results for the RCT. Then, the PP extraction and capsule preparation were done. The details are explained in the following.

#### Clinical intervention

In this phase the RCT was performed. After intervention and data collection, the statistical analysis and conclusion were done.

The details of each phases are explained in the following.

### PHASE 1: Pomegranate peel hydro-alcoholic extraction

#### Dose selection

**Fig. 2:**
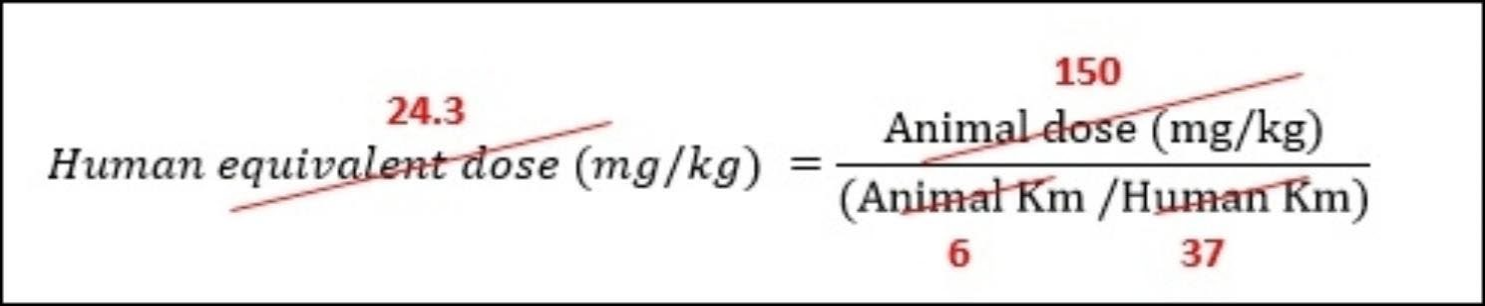
The dose conversion of animal study for RCT: The human equivalent dose was 24.3 mg/kg which for a 60-kg person was about 1500 mg/day

In this study, the dosage of the pomegranate peel supplement was determined based on the research conducted by Wei X. et al. [37] and further confirmed by Wei X.L which demonstrated the effectiveness of the supplement at a dosage of 150 mg/kg in improving liver condition in rats. Under the supervision of the study pharmacologist, the optimal dose conversion from animal to human was calculated using the following formula. The resulting dose was determined to be 1500 mg (Fig. 2). The formula utilized for dose conversion takes into account the body ratio between humans and the studied animal to ensure a safe human dosage that does not induce any complications [38] (Fig. 2). Moreover, previous studies have reported no adverse effects associated with this dosage.

#### Pomegranate peel extraction

The pomegranate peel supplement used in this study was prepared in the Department of Pharmaceutical Sciences in Iranian Medicine, School of Persian and Complementary Medicine, Mashhad University of Medical Sciences, Mashhad, Iran via soaking method [39, 40]. A total of 750 kg of pomegranates of a single variety were procured from the local market in Mashhad. Careful selection was made to choose pomegranates without any chemical, physical, or microbial damage. The pomegranates were then peeled in a pharmacology laboratory and subsequently dried in the shade at a controlled room temperature using an air-conditioned oven. To obtain the extract, a 50% hydro-ethanolic solution was prepared from the dried pomegranate peel powder. The powder was mixed with ethanol (50% v/v), and the resulting suspension was placed on a shaker at 37 °C for 72 to 96 h to extract the active ingredients from the pomegranate peel powder [39]. After 96 h, the liquid was concentrated using a rotary-evaporator device and then dried using a freeze-dryer. The resulting dry powder of the extract was weighed, and the yield of the extract was measured. Standardization of the extract involved measuring the total phenol content using the Folin Miran method, and the extract components were determined using the HPLC method.

#### PP and placebo capsules

For the study’s required daily dose of 1500 mg pomegranate peel (PP) capsules, four capsules of 500 mg each were utilized. Each capsule contained 375 mg of dried extract and an additional 125 mg of microcrystalline cellulose (Avicel®). Therefore, the mixture consisted of 66.6% PP extract and 33.3% Avicel. The pomegranate peel capsules for the intervention group were prepared using a capsule-filling machine. In contrast, the placebo group received capsules filled with 500 mg of placebo, which was prepared using Avicel, food coloring, and a unique essence. The placebo capsules closely resembled the pomegranate peel extract capsules in terms of color, smell, and texture. To maintain blinding, one of the pharmacologic laboratory experts filled the capsules into specific medicine containers under clean conditions. Both groups were labeled as “group A” and “group B,“ and each contained 120 placebo or intervention capsules. The identity of the jars containing the PP capsules was not revealed until the end of the study to ensure blinding.

### PHASE 2: clinical intervention

Upon meeting the eligibility criteria and providing written consent, all participants in this study were divided into control and intervention groups in equal proportions, considering sex and age strata. The intervention group was assigned to receive a daily regimen of four pomegranate peel capsules. Each capsule contained 375 mg of dried peel extract, resulting in a total daily dosage of 1500 mg. The capsules were taken alongside breakfast and dinner for a duration of 8 weeks. Conversely, the control group followed a similar regimen, taking four capsules that contained an inactive ingredient as placebo. Both groups received a 500-kcal deficit diet (53% of carbohydrates, 17% of protein and 30% of fat) along with mediterranean diet advices.

### Data collection

Data were collected at three key time points: baseline, on the 28th day of the intervention, and on the 56th day during the follow-up visit. At baseline, participants completed a questionnaire to gather demographic characteristics. Anthropometric assessments, including height, weight, body mass index (BMI), body composition, blood pressure, and gastrointestinal complications, were measured at baseline, in the middle, and at the end of the intervention.

To assess hepatic status, transient elastography was performed at beginning and the end of the study. Participants also completed a 3-day food record and the International Physical Activity Questionnaire (IPAQ) at baseline and at the end of the intervention to evaluate dietary intake and physical activity levels, respectively. Moreover, biochemical variables, including complete blood count with differential (CBC-diff), lipid profiles including low-density lipoprotein (LDL-C), high-density lipoprotein (HDL-c), triglyceride (TG), and total cholesterol (TC), fasting blood sugar (FBS) and serum insulin, were assessed at baseline and at the end of the study.

### Statistical analysis

The data collected in this study were analyzed using version 16 of the SPSS statistical software. Intention-to-treat analysis was employed to accurately estimate the intervention effect, with missing data imputed using the last observed value. The distribution of qualitative variables both among and within groups was assessed using the chi-square test. For comparisons between groups of quantitative variables with a normal distribution, the independent samples t-test was used, while the paired t-test was utilized for comparisons within groups over time. In cases where the distribution of variables was non-normal, the Mann-Whitney U test and Wilcoxon signed-rank test were employed for between-group and within-group comparisons, respectively. To minimize potential confounding effects of variables such as baseline values, changes in energy intake, physical activity, and body weight on secondary outcomes, analysis of covariance (ANCOVA) was performed. Qualitative variables were presented as percentages, quantitative variables with a skewness of less than one were reported as mean ± standard deviation, and variables with a skewness equal to or greater than one were presented as median (interquartile range). P values of less than 5% (p < 0.05) was considered statistically significant.

## Results

**Fig. 3:**
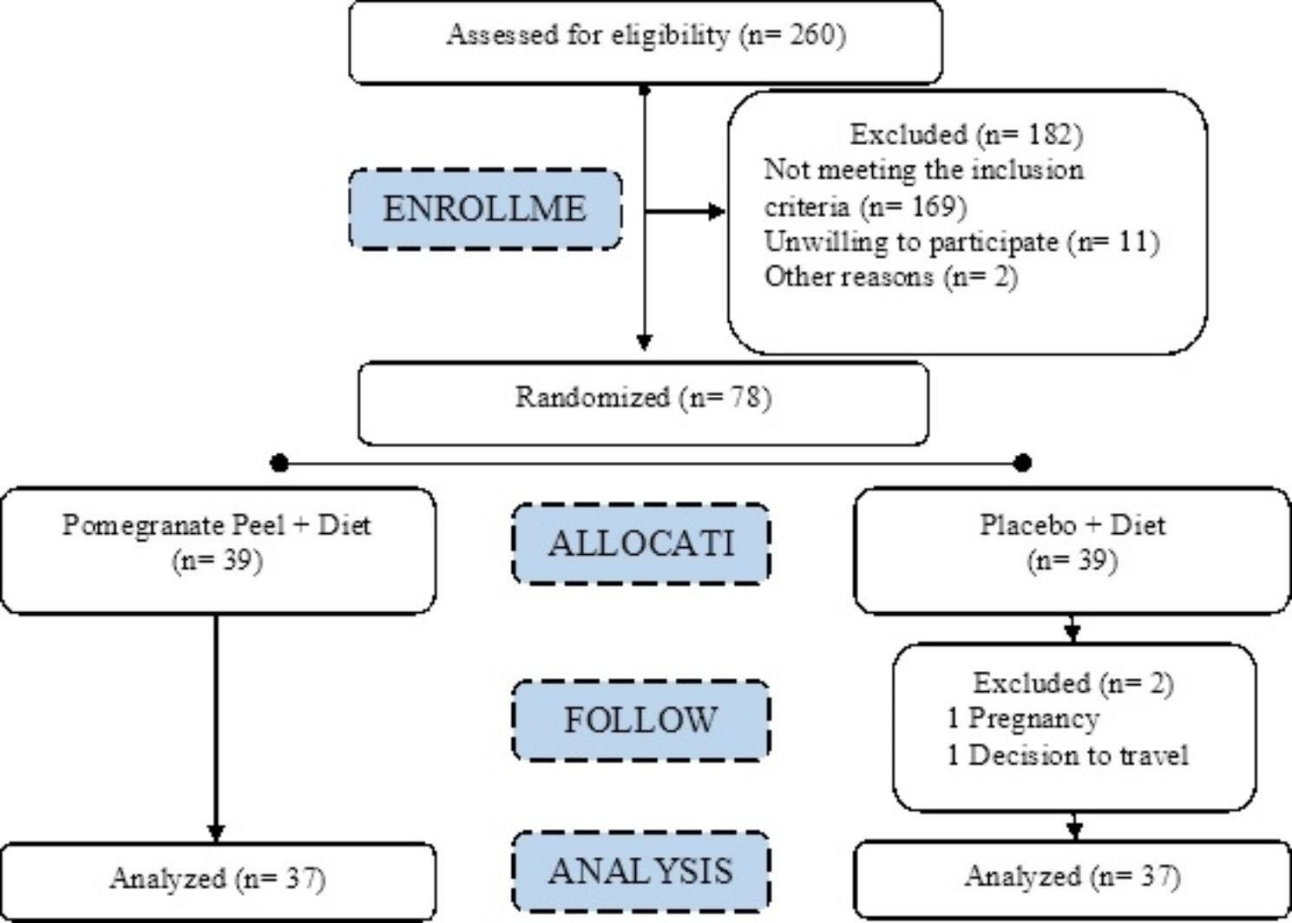
The flow diagram of participant selection process and study procedure

The study was conducted between December 2021 and the spring, summer, and fall of 2022, and it involved adult patients diagnosed with non-alcoholic fatty liver disease who met the predetermined inclusion criteria. Prior to their participation, written informed consent was obtained from all individuals. A total of 78 participants were enrolled and randomly allocated to either the pomegranate peel group or the placebo group, utilizing a stratified block design to ensure equal distribution. Specifically, 39 participants were assigned to the pomegranate peel group, while the remaining 39 were assigned to the placebo group. The participants were then observed and followed up for a duration of 8 weeks. During the study period, the majority of patients adhered to their assigned treatment, with only two individuals from the placebo group discontinuing their participation. The flow diagram of participant selection process and study procedure is illustrated in Fig. 3.

Gastrointestinal symptom severity rating (GSSR) did not change significantly within (P = 0.29 and 0.54, respectively) and also between (P = 0.64) pomegranate peel and placebo group when measured in different time sequences. The quality assessment of the extract reported the mean polyphenol content (ellagic acid)/gram of 15%.

### Baseline characteristics

The results of demographic data of the study participants by study groups are presented in Table 1. The results of the study indicate that the randomization process was effectively implemented, ensuring a balanced distribution of participants across the study groups. The mean age of the participants was 43.1 ± 8.6 years, with no statistically significant difference between the pomegranate peel group (mean age 42.8 ± 7.2 years) and the placebo group (mean age 43.3 ± 10.13 years) (p = 0.8). Out of the enrolled participants, 37 (48.7%) were male and 39 (51.3%) were female, and there was no significant difference in sex distribution between the groups (p = 0.65). Regarding body weight status, among the participants in the pomegranate peel group, 6.2% were classified as normal weight, 35.9% as overweight, and 61.5% as obese. In the placebo group, the corresponding percentages were 7.2%, 37.8%, and 54.9% for underweight, overweight, and obese categories, respectively. However, there was no significant difference in body weight status between the two groups (p = 0.15). Furthermore, no significant differences were observed between the pomegranate peel group and the placebo group in terms of other demographic variables, as indicated in the Table 1.

**Table 1:** Baseline characteristics of study participants

Variable		PP	Placebo	P _value_
**Age (years)**		42.8 ± 7.2	43.3 ± 10.1	0.81 *
**Women**		21 (53.8)	18 (48.6)	0.65
**Type 2 diabetes**		3 (7.7)	3 (8.1)	0.99
**Cardiovascular disease**		5 (12.8)	3 (8.1)	0.71
**Hyperlipidemia**		13 (33.3)	12 (32.4)	0.62
**Hypertension**		6 (15.4)	6 (16.2)	0.99
**Smoking and others**		5 (12.8)	6 (16.2)	0.67
**Physical Activity**	Low	19 (48.7)	18 (48.6)	0.99
	Medium	19 (48.7)	18 (48.6)	
	High	1 (2.6)	1 (2.7)	
**BMI Classification** **(kg/m** ^**2**^ **)**	Underweight	0 (0)	1 (2.7)	0.15
	Normal	1 (2.6)	0 (0)	
	Overweight	14 (35.9)	22 (59.5)	
	Obese	24 (61.5)	14 (37.8)	

Data is presented as Mean ± SD or Number (percent).

P extracted of Chi Square except (*), which is obtained from 2 independent sample t test

### Dietary intake evaluations

Table 2 in the study presents the results of dietary intake evaluations for both the pomegranate peel and placebo groups at the beginning and end of the study. The table indicates the mean food intake in each group and examines the differences between them. At baseline, there was no significant difference observed between the two groups (P > 0.05). During the study, following the prescribed dietary regime and supplement intake, both the pomegranate peel and placebo groups showed significant reductions in mean energy, carbohydrate, and saturated fat intakes (p < 0.05). However, when comparing the mean changes in nutrient intakes between the two groups, no statistically significant differences were found. To provide a more comprehensive analysis, the mean changes in nutrient intake were reported as both raw values and energy-adjusted values. Moreover no significant changes was observed between groups in mean dietary vitamin E intake (-1.95 ± 8.90 in the placebo and − 2.52 ± 6.70 mg in the PP groups) as a representative of antioxidant intake from diet, during the study (P = 0.79).

**Table 2:** Dietary intake changes of placebo and PP groups during the study

Variables	Study groups	Before	P _value_	After	P _value_*	Changes	P _value_**
Energy Kcal	Placebo	2524.83 ± 318.82	0.39	1899.27 ± 301.46	< 0.001	-625.56 ± 131.38	0.56
	PP	2463.74 ± 264.10		1820.51 ± 264.75	< 0.001	-643.23 ± 133.53	
Protein (gr)	Placebo	87.90 ± 15.11	0.85	82 ± 21.10	0.26	-5.80 ± 26.9	0.94
	PP	89.01 ± 24.10		82.60 ± 16.50	0.22	-6.40 ± 26.5	
Carbohydrate (gr)	Placebo	352.20 ± 59.30	0.15	317.90 ± 61.40	0.01	-34.30 ± 66.30	0.92
	PP	374.50 ± 52.60		341.50 ± 61.10	0.001	-32.90 ± 37	
Fat (gr)	Placebo	71.09 ± 25.90	0.64	66.45 ± 23.30	0.38	-4.60 ± 27.10	0.80
	PP	73.80 ± 22.05		67.30 ± 19.70	0.20	-6.50 ± 22.80	
Fiber (gr)	Placebo	18.70 ± 5.70	0.77	16.20 ± 6.30	0.12	− 2.54 ± 8.30	0.35^&^
	PP	18.37 ± 4.10		17.70 ± 4.60	0.54	-0.69 ± 5.90	
Cholesterol (mg)	Placebo	227.2 ± 105	0.67	217.1 ± 109	0.68	-11.01 ± 135	0.62
	PP	236.31 ± 91.12		211.05 ± 103.35	0.28	-25.10 ± 118.05	
Saturated fat (gr)	Placebo	21.87 ± 8.80	0.72	19.37 ± 6.80	0.07	-2.50 ± 6.90	0.13
	PP	22.73 ± 8.70		18.05 ± 8.90	0.002	-5.70 ± 8.40	
Vitamin E (mg)	Placebo	8.64 ± 7.15	0.45	6.70 ± 4.40	0.27^#^	-1.95 ± 8.90	0.79^&^
	PP	9.96 ± 5.60		8.40 ± 4.10	0.06^#^	-2.52 ± 6.70	

Data is presented as Mean ± SD.

P and P^**^ is extracted from 2 independent sample t test, P * is extracted from Paired t test, unless those with & and # that are extracted from Mann-Whitney and Wilcoxon, respectively.

Changes, represent before and after changes in raw model.

gr: Grams; mg: Milligrams; and PP is for the Pomegranate Peel group.

### Body composition evaluations

The results of the body composition assessments during the study for each study group are presented in Table 3. In this table, the raw mean and adjusted model based on baseline values, weight, energy, and physical activity for each variable are reported. As presented in table, there was no significant difference observed between the two groups at baseline (P > 0.05). Following the intervention, the mean body weight, waist circumference, body mass index, body fat index, and trunk fat decreased in both the pomegranate peel and placebo groups. Although these changes did not differ significantly between the two groups statistically, at beginning the study. However, comparing different time sequences, weight (-0.62 ± 1.08 and − 5.10 ± 2.30 kg in the placebo and PP groups, respectively), waist circumferences (WC) (-1.05 ± 1.68 and − 7.57 ± 2.97 cm in the placebo and PP groups, respectively), fat free mass (+ 0.01 ± 0.01 and − 0.92 ± 0.90 kg in the placebo and PP groups, respectively), fat mass index (-0.21 ± 0.15 and − 1.49 ± 0.86 in the placebo and PP groups, respectively), total body water (+ 0.51 ± 0.16 and + 2.35 ± 1.83% in the placebo and PP groups, respectively), trunk fat (− 0.64 ± 0.25 and − 3.93 ± 3.07% in the placebo and PP groups, respectively) decreased significantly more in the pomegranate peel group than in the placebo group (All P < 0.001 except for fat free mass (P < 0.003)). Moreover, after adjusting for potential confounders, physical activity, and energy intake, the mean changes remained significantly greater in the pomegranate peel group than in the placebo group.

**Table 3:** Anthropometric changes of placebo and PP groups during the study

	Study Groups	Before	P*	Middle	After	Changes	P Group	P time	P Group x time	P **
Weight (kg)	Placebo	90 ± 14.18	0.84	89.43 ± 14.24	89.38 ± 14.32	-0.62 ± 1.08	0.38	< 0.001	< 0.001	< 0.001
	PP	89.56 ± 12.06		86.02 ± 13.51	84.46 ± 12.91	-5.10 ± 2.30				
WC (cm)	Placebo	107.59 ± 8.36	0.11	106.95 ± 8.90	106.54 ± 8.66	-1.05 ± 1.68	0.94	< 0.001	< 0.001	< 0.001
	PP	111.19 ± 10.4		106.71 ± 11.63	103.61 ± 10.8	-7.57 ± 2.97				
BMI (kg/m^2^)	Placebo	30.38 ± 4.29	0.25	30.21 ± 4.37	30.17 ± 4.37	-0.20 ± 0.04	0.76	< 0.001	< 0.001	< 0.001
	PP	31.5 ± 4.21		30.47 ± 4.21	29.67 ± 4.27	-1.82 ± 0.85				
Fat mass index	Placebo	8.29 ± 3.59	0.11	7.95 ± 3.72	8.07 ± 3.63	-0.21 ± 0.15	0.33	< 0.001	< 0.001	< 0.001
	PP	9.58 ± 3.51		8.97 ± 3.39	8.09 ± 3.36	-1.49 ± 0.86				
Body water (%)	Placebo	52.85 ± 6.34	0.07	53.81 ± 6.70	53.37 ± 6.43	+ 0.51 ± 0.16	0.23	< 0.001	< 0.001	< 0.001
	PP	50.30 ± 5.63		50.30 ± 5.57	52.65 ± 5.76	+ 2.35 ± 1.83				
Fat Free Mass (kg)	Placebo	65.79 ± 10.55	0.19	66.23 ± 10.42	65.80 ± 10.16	+ 0.01 ± 0.01	0.09	0.004	0.003	< 0.001
	PP	62.79 ± 9.64		61.64 ± 10.11	61.89 ± 9.46	− 0.92 ± 0.90				
Trunk Fat (%)	Placebo	26.26 ± 7.81	0.43	24.95 ± 8.48	25.62 ± 8.10	− 0.64 ± 0.25	0.84	< 0.001	< 0.001	< 0.001
	PP	27.63 ± 7.43		26.56 ± 7.22	23.7 ± 8.55	− 3.93 ± 3.07				

Data is presented as Mean ± SD.

P* is extracted from 2 independent sample t test, and P** is presented adjusted model by baseline values, energy intake and physical activity, with ANCOVA. Also, P for group, time and group x time is extracted from repeated measures for raw model.

“Changes” represent before and after changes in raw model.

BMI: Body Mass Index, cm: centimeters; kg: kilograms; m: meters; WC: Waist Circumferences; and PP is for Pomegranate Peel group.

### Lipid and glycemic profile evaluations

The results of serum metabolic indices assessment during the study for each study group are presented in Table 4. In this table, the raw mean and adjusted model based on baseline values, weight, energy, and physical activity for each variable are reported. As presented in table there was no significant difference observed between the two groups at baseline (P > 0.05).

In this study, following the diet plan and supplemental interventions, fasting blood glucose levels (FBS) (0.32 ± 0.07 and − 5.28 ± 1.36 mg/dl in the placebo and PP groups, respectively), triglyceride to glucose ratio (-0.008 ± 0.11 and − 0.16 ± 0.11 in the placebo and PP groups, respectively), total cholesterol (TC) (3.21 ± 0.95 and − 10.51 ± 0.77 mg/dl in the placebo and PP groups, respectively), triglyceride (TG) (0.27 ± 0.96 and − 16.02 ± 1.7 mg/dl in the placebo and PP groups, respectively), low-density lipoprotein-cholesterol (LDL-C) (1.40 ± 2.90 and − 9.33 ± 6.66 mg/dl in the placebo and PP groups, respectively), and cholesterol to lipoprotein ratio (0.009 ± 0.21 and − 0.74 ± 0.73 in the placebo and PP groups, respectively) were significantly decreased in pomegranate peel group (All P < 0.001 except from FBS (P = 0.02)). Additionally, serum HDL-C levels (0.70 ± 0.18 and 5.10 ± 0.36 mg/dl in the placebo and PP groups, respectively; P < 0.001) were significantly increased in individuals who received the pomegranate peel. Moreover, after adjusting for potential confounders, physical activity, and energy intake, the mean changes remained significantly greater in the pomegranate peel group than in the placebo group. Changes in fasting insulin (2.78 ± 1.57 and 1.73 ± 2.27 µIU/ml in the placebo and PP groups, respectively; P = 0.81) and homeostatic model assessment for insulin resistance (HOMA-IR) (0.39 ± 0.68 and 0.83 ± 1.20 in the placebo and PP groups, respectively; P = 0.93) were not significantly different when comparing two groups during the study in raw and even adjusted model.

**Table 4:** Lipid and glycemic profile changes of placebo and PP groups during the study

Variables	Group	Before	P _value_	After	P _value_*	Changes	P _value_**	P _value_#
FBS (mg/dl)	Placebo	102.56 ± 20.87	0.10	102.89 ± 20.40	0.78	0.32 ± 0.07	0.02	0.02
	PP	96.28 ± 9.90		91.15 ± 55	0.02	-5.28 ± 1.36		
Insulin (µIU/ml)	Placebo	19.14 ± 11.35	0.83	21.92 ± 18.12	0.29	2.78 ± 1.57	0.81	0.93
	PP	19.57 ± 6.17		21.30 ± 23.31	0.63	1.73 ± 2.27		
HOMA-IR	Placebo	4.95 ± 3.05	0.63	5.64 ± 4.60	0.29	0.39 ± 0.68	0.93	0.87
	PP	4.68 ± 1.83		5.51 ± 10.38	0.61	0.83 ± 1.20		
TG/FBS index	Placebo	8.50 ± 0.49	0.02	8.50 ± 0.52	0.65	-0.008 ± 0.11	< 0.001	< 0.001
	PP	8.83 ± 0.38		8.66 ± 0.35	< 0.001	-0.16 ± 0.11		
TC (mg/dl)	Placebo	172.59 ± 23.05	0.57	175.80 ± 21.53	0.055	3.21 ± 0.95	< 0.001	< 0.001
	PP	175.66 ± 24.52		165.15 ± 22.74	< 0.001	-10.51 ± 0.77		
TG (mg/dl)	Placebo	108.64 ± 49.80	0.06	109.1 ± 47.60	0.86	0.27 ± 0.96	< 0.001	< 0.001
	PP	151.38 ± 49.90		135.35 ± 42.20	< 0.001	-16.02 ± 1.7		
HDL-C (mg/dl)	Placebo	41.70 ± 5.36	0.68	42.70 ± 5.36	0.02	0.70 ± 0.18	< 0.001	< 0.001
	PP	42.28 ± 6.95		47.38 ± 7.02	< 0.001	5.10 ± 0.36		
LDL-C (mg/dl)	Placebo	89.48 ± 12.59	0.06	90.89 ± 12.73	0.16	1.40 ± 2.90	< 0.001	< 0.001
	PP	95.82 ± 14.6		86.48 ± 11.32	< 0.001	-9.33 ± 6.66		
Chol/HDL-C	Placebo	4.16 ± 0.49	0.53	4.17 ± 0.45	0.79	0.009 ± 0.21	< 0.001	< 0.001
	PP	4.29 ± 1.24		3.55 ± 0.76	< 0.001	-0.74 ± 0.73		

Data is presented as Mean ± SD.

P and P** are extracted from 2 independent sample t test. P* is extracted from paired t test for raw model. Also, P# is presented adjusted model by baseline values, and potential confounders including weight, energy intake and physical activity, with ANCOVA.

“Changes” represent before and after changes in raw model; “PP” is for Pomegranate Peel group.

Chol: Cholesterol, dl: Deciliter, FBS: Fasting Blood Sugar, HDL-C: High Density Lipoprotein- Cholesterol, HOMA-IR: Homeostatic Model Assessment for Insulin Resistance, µIU: Micro International Unit, mg: Milligrams, ml: Milliliter, LDL-C: Low Density Lipoprotein- Cholesterol, TC: Total Cholesterol, TG: Triglyceride,

### Blood pressure evaluations

The results of Systolic and diastolic blood pressure values during the study for each study group are presented in Table 5. In this table, the raw mean and adjusted model based on baseline values, weight, energy, and physical activity for each variable are reported. There were a significant difference between the two groups at baseline in systolic blood pressure (SBP) (P = 0.01) but not in diastolic blood pressure (DBP) (P = 0.05). Although the mean systolic and diastolic blood pressures decreased in both the pomegranate peel and control groups, the reductions were not statistically significant (P = 0.65 and 0.23, respectively). Nevertheless, the slope of the changes in the pomegranate peel group was significantly steeper than that of the control group in the measured time sequences in systolic (-0.16 ± 0.19 and − 0.63 ± 0.29 cmHg in the placebo and PP groups, respectively; P < 0.001) and diastolic pressure (-0.09 ± 0.16 and − 0.39 ± 0.19 cmHg in the placebo and PP groups, respectively; P < 0.001).

**Table 5:** Blood pressure changes of placebo and PP during the intervention

Variables		Before	P *	Middle	After	Changes	P Group	P time	P Group x time	P **
Systolic Blood Pressure (cmHg)	Placebo	13.50 ± 1.21	0.01	13.40 ± 1.14	13.30 ± 1.15	-0.16 ± 0.19	0.65	< 0.001	< 0.001	< 0.001
	PP	14.30 ± 1.48		13.90 ± 1.44	13.60 ± 1.40	-0.63 ± 0.29				
Diastolic Blood Pressure (cmHg)	Placebo	8.50 ± 0.74	0.05	8.50 ± 0.70	8.40 ± 0.70	-0.09 ± 0.16	0.23	< 0.001	< 0.001	< 0.001
	PP	8.90 ± 0.76		8.70 ± 0.77	8.50 ± 0.75	-0.39 ± 0.19				

Data is presented as Mean ± SD.

P* is extracted from 2 independent sample t test, and P** is presented adjusted model by baseline values, energy intake and physical activity, with ANCOVA. Also, P for group, time and group x time is extracted from repeated measures for raw model.

“Changes” represent before and after changes in raw model. “PP” is for Pomegranate Peel group. cmHg: Centimeters of Mercury

### Fatty liver status evaluations

Table 6 presents the liver elastography results for the pomegranate peel and placebo groups at baseline and the end of the study in raw and adjusted model. There were no significant differences between the groups at baseline in liver stiffness and hepatorenal ultrasound index (p = 0.06 and 0.07, respectively). At the end of the study, both groups showed a significant reduction in liver elastography reports in within group analysis. However, when comparing between two groups, the pomegranate peel group exhibited a significantly greater decrease in liver stiffness (0.14 ± 0.02 kPa in the placebo in contrast to -0.72 ± 0.35 kPa in the PP group; P < 0.001) and hepatorenal ultrasound index (0.07 ± 0.05 in the placebo in contrast to − 0.30 ± 0.17 in the PP group; P < 0.001) in raw and even after adjusting for baseline values and potential confounders. The improvements in the pomegranate peel group was statistically significant (P = 0.002). However, the changes in the degree of liver steatosis in the control group were not statistically significant (P = 0.66). The results of the between-group analysis showed that the reduction in the degree of liver steatosis in the pomegranate peel group was statistically significant compared to the control group, even after adjusting for potential confounding factors (P < 0.001).

**Table 6:** Liver elastography results of placebo and PP during the intervention

Variables	Study group	Before	P _value_	After	P _value_*	Changes	P _value_**	P _value_#
Liver stiffness (Kilo Pascal)	Placebo	5. 1 ± 0.56	0.07	5.24 ± 0.55	< 0.001	0.14 ± 0.02	< 0.001	< 0.001
	PP	5.43 ± 0.39		4.71 ± 0.47	< 0.001	-0.72 ± 0.35		
Hepatorenal ultrasound index	Placebo	1.82 ± 0.24	0.06	1.9 ± 0.27	0.03	0.07 ± 0.05	< 0.001	< 0.001
	PP	1.98 ± 0.25		1.66 ± 0.19	< 0.001	− 0.30 ± 0.17		

Data is presented as Mean ± SD.

P and P** are extracted from 2 independent sample t test. P* is extracted from paired t test for raw model. Also, P^#^ is presented adjusted model by baseline values, and potential confounders including weight, energy intake and physical activity, with ANCOVA.

“Changes” represent before and after changes in raw model; “PP” is for Pomegranate Peel group.

## Discussion

**Fig. 4:**
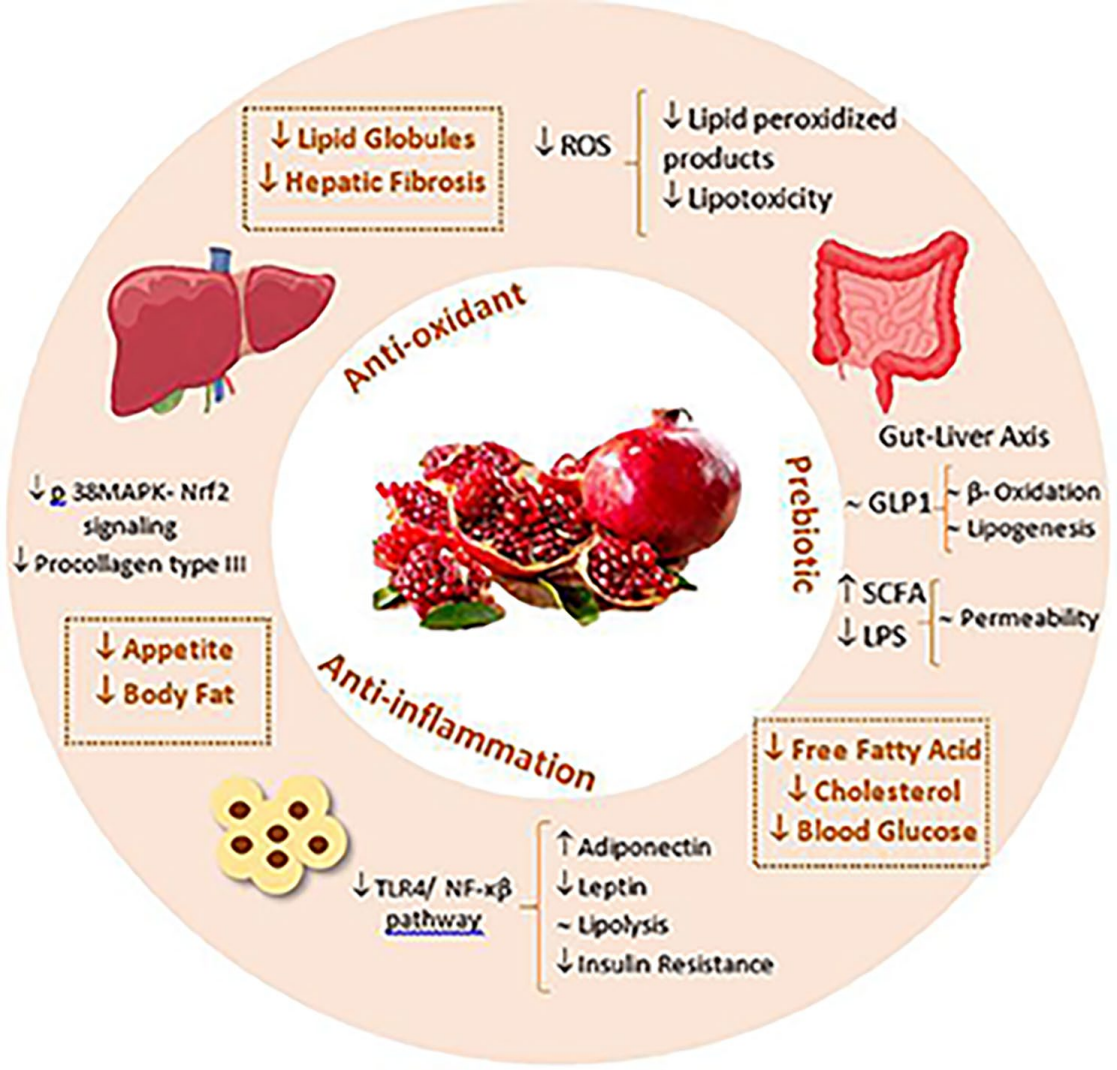
Effects of pomegranate peel on metabolic syndrome risk factors. In this figure, the anti-oxidant, anti-inflammatory, and prebiotic effects of pomegranate peel are illustrated. PP as a prebiotic leads to the production of short-chain fatty acids (SCFA), the reduction of lipopolysaccharides (LPS), and the regulation of GLP-1 secretion. On the other hand, PP resulted in reduced inflammatory pathways and regulated lipolysis and insulin sensitivity. Together, they cause lower cholesterol and blood sugar levels, as well as lower fibrosis in the liver. Also, PP as an antioxidant leads to lower lipotoxicity and regulated lipid-related hormones such as leptin and adiponectin which all result in lower lipid globules in the liver and reduced appetite as well as weight gain

Our results suggested that an 8-week pomegranate peel supplementation along with a 500-calorie deficit diet, resulted in improvement of liver steatosis grade, as well as metabolic syndrome risk factors including weight, body fat index, metabolic biochemistry indices, and blood pressure of NAFLD patients in contrast to placebo. In the following, we will discuss about effects of pomegranate peel (PP) on risk factors of metabolic syndrome (MS) including anti-obesity, anti-glycemic, anti-hypertension, lipid lowering, and fatty liver improvement effects. Figure 4 summarized the effects of PP on MS risk factors.

### Anti-obesity and anti-fat effects of pomegranate peel

The study findings suggest that pomegranate peel supplementation significantly reduces weight, body mass index, waist circumference, fat mass index, and trunk fat in patients with non-alcoholic fatty liver disease after adjusting for potential confounders and baseline values. Furthermore, in adjusted model, the pomegranate peel group showed a statistically significant decrease in fat free mass, with a mean change of -0.99 ± 2.0 kg. In comparison, the control group showed a tiny increase of 0.08 ± 2.0 kg. The decrease in the FFM in the pomegranate peel (PP) group may be due to the lack of control over all factors and confounding variables affecting FFM such as diet, physical activity, age and sex, race, and hormonal status of the individual.

The study conducted by Grabez et al. [18] was consistent with our findings regarding decreased waist circumference in the group receiving pomegranate peel. However, they did not find significant differences in weight, body mass index, body fat percentage, or fat free mass index. Similarly, Khadem-Haghighian et al. [41] did not observe a significant difference in mean BMI changes before and after intervention in osteoarthritis patients receiving pomegranate peel.

A review of changes in anthropometric indices between studies revealed conflicting results in animal studies [42–44]. A study conducted on rats with a high-fat diet in 2019 found that the group receiving cake containing 15% pomegranate peel powder had less weight gain than other groups [42], consistent with our findings. However, evidence suggests that the appetite-reducing effects of pomegranate peel are greater under high-fat diet conditions. Another study by Soliman and colleagues in 2022 on overweight rats reported a significant decrease in leptin levels with pomegranate peel powder supplementation[43]. However, Rahnama et al. reported in their study on obese desert rats, who had two months of aerobic exercise and pomegranate peel consumption, that pomegranate peel extract with exercise led to a 50% decrease in body weight [44]. They did not observe a significant effect on rats’ weight or insulin levels.

In our study, we observed no significant impact of the pomegranate peel extract supplement on the dietary intake of the participants. These findings align with previous investigations, such as the study by Khadem-Haghighian et al.[45], which examined the effects of pomegranate peel extract on overweight women with knee osteoarthritis. Similarly, Grabez et al. [18], explored the effects of pomegranate peel in diabetic patients and did not report any substantial alterations in energy intake before and after the intervention. Correspondingly, a study involving overweight women with dyslipidemia [41] and another study on hemodialysis patients by Jafari et al. [46] also did not find any notable changes in macronutrient and micronutrient intake following pomegranate peel supplementation. Therefore, collectively, the published clinical trials on pomegranate peel support our findings.

Although the reduction in calorie intake in the pomegranate peel group may not have reached statistical significance compared to the control group, it is noteworthy from a nutritional standpoint as it contributed to greater weight loss. Animal studies have indicated that pomegranate peel can positively affect appetite through its influence on leptin [43, 44]. Hence, the underreporting of meal intake by individuals who did not achieve weight loss could potentially account for the lack of difference in dietary intake between the two study groups. Our study advised all patients to have an active lifestyle. However, there was no changes in physical activity levels before and after the intervention in either group. The difference in our findings compared to other studies may be due to the fact that in our study, a calorie-restricted diet and recommendations for a Mediterranean diet were provided to both groups for ethical reasons. However, weight, fat mass, and FFM did not show significant changes in the control group. The mechanism of pomegranate peel’s effect on weight and appetite changes may occur through inhibition of the pancreatic lipase enzyme, suppression of energy intake, and a significant reduction in leptin levels [43, 44]. The appetite-reducing effects of pomegranate peel can explain the lack of significant weight loss in the control group despite receiving a similar low-calorie diet during the study and a weaker diet acceptance than the intervention group.

### Anti-glycemic effects of pomegranate peel

The results of our study indicate that daily supplementation of 1500 milligrams of pomegranate peel for 8 weeks significantly reduces fasting blood glucose levels, even after adjusting for potential confounding factors. However, our intervention did not result in a significant change in fasting serum insulin levels. Additionally, our results showed that pomegranate peel supplementation improves the triglyceride-glucose index, which measures insulin resistance but does not significantly affect the homeostatic model assessment of insulin resistance (HOMA-IR). In a similar study by Grabez et al.[18], pomegranate peel supplements were administered to patients with type 2 diabetes for 8 weeks. However, no significant changes were observed in fasting blood glucose levels and insulin sensitivity, including the HOMA-IR and QUICKI indices. However, a significant difference in the level of hemoglobin A1C was reported. Furthermore, consistent with our study, there was no significant difference in serum insulin levels between the pomegranate peel and placebo groups. The different results in fasting blood glucose levels compared to our study may be due to the fact that participants in the Grabez study [18] had diabetes and were receiving routine and long-term metformin therapy, as well as the lack of dietary recommendations in that study.

Animal studies on pomegranate peel have shown contradictory results [47–50]. While pomegranate peel extract has been shown to significantly reduce serum glucose levels in diabetic rats with or without induced diabetes [47–49], another animal study on rats with induced diabetes did not report any significant effects of pomegranate peel supplementation on weight or blood glucose levels [50]. In most studies, the intervention dose was lower than in our study, and diabetic samples were used, which may explain the contradictory results. Animal studies have shown that pomegranate peel’s polyphenols can improve carbohydrate metabolism and insulin sensitivity by inhibiting alpha-glucosidase and alpha-amylase enzymes, reducing blood glucose levels [51, 52].

### Lipid lowering effects of pomegranate peel

One of the significant findings of our study was a meaningful reduction in the levels of total cholesterol, triglycerides, low-density lipoprotein (LDL), and a significant increase in the levels of high-density lipoprotein (HDL) in the pomegranate peel group compared to the placebo group. Our results are consistent with the study by Khadem-Haghighian et al.[41], who investigated the effects of a pomegranate peel extract supplement (1000 mg/day) for 8 weeks on lipid profiles in obese women with dyslipidemia in 2016. They reported a significant improvement in total cholesterol, triglycerides, and LDL cholesterol levels. However, the trend toward reduction in the lipid profile in our study was greater, which may be due to the difference in intervention dosage between the two studies.

In another study conducted by Grabez et al. [18] in 2019, pomegranate peel supplement was administered to patients with type 2 diabetes for 8 weeks. Similar to our study, they reported a significant reduction in serum triglyceride levels, the ratio of LDL to HDL cholesterol, and a significant increase in high-density lipoprotein cholesterol levels. However, there was no significant difference in total cholesterol and LDL levels. Another study in 2021 administered a pomegranate peel supplement (1000 mg/day) for 8 weeks to women with osteoarthritis, which resulted in a significant reduction in total cholesterol and triglyceride levels[45], which is consistent with our results. However, there was no significant difference in serum levels of high-density and low-density lipoproteins. They attributed the lack of significance in the pomegranate peel group’s lipid profile changes to the high standard deviation of the serum lipoprotein levels, which could potentially be resolved by increasing the sample size.

Moreover, animal studies have reported pomegranate peel’s cholesterol and triglyceride-lowering effects [53, 54], which aligns with our findings. The conflicting results regarding the effect of pomegranate peel on lipid profiles may be due to the small sample size, different treatment durations, supplement dosages, and types of pomegranates used in supplement production.

Furthermore, in our study, the ratio of cholesterol to high-density lipoprotein cholesterol significantly decreased by 72.0 ± 0.5% in the pomegranate peel group compared to the placebo group. A ratio below 5 is acceptable, but values less than 3.5 are considered excellent [55]. Therefore, our study demonstrated that a pomegranate peel supplement of 1500 mg/day for 8 weeks have beneficial effects on the serum lipid profile in patients with fatty liver, as the cholesterol to high-density lipoprotein cholesterol ratio decreased from 24.1 ± 29.4 to 0.76 ± 55.3, which is close to the acceptable range. These results are valuable in evaluating PP effects on cardiovascular disease risk of NAFLD patients which are due to pomegranate peel’s anti-inflammatory, antioxidant, lipid-lowering, and blood pressure-lowering effects and also its constituent compounds, such as ellagic acid.

The lipid-lowering effects of pomegranate peel are related to its polyphenolic compounds, such as ellagic acid, gallic acid, punicalagin, and ellagic acid tannins [56–59]. Studies have shown that these polyphenols inhibit the activity of pancreatic lipase, 3-hydroxy-3-methylglutaryl-coenzyme A (HMG-CoA) reductase, and acyl-CoA: cholesterol acyl transferase (ACAT), resulting in a decrease in serum cholesterol and triglyceride levels [56, 57]. Also, catechins present in pomegranate peel reduce intestinal fat absorption and inhibit critical enzymes in lipid biosynthesis [58]. Another polyphenolic compound found in pomegranate peel is ellagic acid, which has lipid-lowering effects by activating peroxisome proliferator-activated receptor gamma (PPAR) receptor and increasing cholesterol metabolism in L02 cells, a human hepatocyte cell model. This can improve the underlying causes of cardiovascular diseases [59].

### Anti-hypertension effects of pomegranate peel

In our study, systolic and diastolic blood pressure were evaluated at baseline, midpoint, and endpoint of the intervention. The pomegranate peel group showed a significant decrease in systolic and diastolic blood pressure even after adjusting for potential confounding factors.

Khadem-Haghighian et al. [41] investigated the effect of a supplement containing 1000 mg of pomegranate peel extract for 8 weeks in 38 obese women with dyslipidemia. After the intervention, a significant decrease was observed in the systolic blood pressure of the patients, which is consistent with our results. However, the mean diastolic blood pressure did not change significantly despite the decreasing trend. This difference in results may be due to the sample size in the two studies. As a decreasing trend in diastolic blood pressure was observed in their study, statistical significance may be achieved with an increase in sample size. Grabez et al. (2019) [18] reported a significant decrease in systolic and diastolic blood pressure after receiving 1000 mg of dried pomegranate peel extract for 8 weeks in 38 diabetic patients, which is consistent with our results. Studies on pomegranate juice, which contains some similar polyphenols to pomegranate peel, have also confirmed the antihypertensive effects of pomegranate on systolic and diastolic blood pressure [19, 24, 25]. The probable mechanism is related to the polyphenols present in pomegranate peel and fruit, which lead to a reduction in the level of angiotensin-converting enzyme and improvement of vasodilation through the effect on nitric oxide [60–62].

### Anti-steatosis and anti-fibrosis effects of pomegranate peel

Our study demonstrated a significant reduction in liver stiffness and the hepatorenal ultrasound index among patients receiving pomegranate peel supplements. In contrast, the placebo group exhibited a slight increase in these indices, which reached statistical significance. This discrepancy may be attributed to non-adherence to the recommended diet and physical activity in some individuals within the placebo group. Notably, no clinical trial investigating the effects of pomegranate peel on patients with non-alcoholic fatty liver disease has been published thus far. Therefore, our study relied on existing animal and human studies in this field.

The findings of our study align with previous research examining the impact of pomegranate peel extract on liver fibrosis and fatty liver disease in animal models. For instance, Vi et al. administered pomegranate peel extract to rats with carbon tetrachloride-induced liver fibrosis for a duration of four weeks in 2015. They observed a reduction in aspartate and alanine transaminases, as well as total bilirubin levels. Additionally, they reported improvements in liver fibrosis by reducing markers such as hydroxyproline, procollagen type III, hyaluronic acid, laminin, and TGF-β1 [19]. Another study investigating liver fibrosis examined rats with obstructed bile ducts who were treated with 50 mg/kg body weight of pomegranate peel extract for a duration of 13 weeks. The results revealed notable improvements in various liver-related parameters, including reductions in aspartate and alanine transaminases, lactate dehydrogenase levels, and liver myeloperoxidase activity. Furthermore, there was an enhancement in liver antioxidant capacity and a decrease in malondialdehyde levels compared to the control group. These findings suggest that pomegranate peel extract may have a beneficial effect on liver fibrosis and associated liver markers in this animal model [63].

Also, our findings align with the research conducted by Liu et al., who demonstrated a reduction in liver fat content and inflammation following a 13-week administration of pomegranate peel extract in mice [64]. Similarly, Abou-Zeid et al. observed a decrease in cholesterol and triglyceride levels in rat liver tissue after a 30-day intervention [65]. Several other animal studies investigating the effects of pomegranate peel on fatty liver disease have reported similar outcomes to our study [66–68]. These consistent findings across multiple animal studies further support the potential beneficial effects of pomegranate peel in ameliorating fatty liver disease.

Pomegranate peel has been attributed with various mechanisms underlying its anti-steatotic effects. These mechanisms encompass the enhancement of adiponectin signaling, modulation of genes involved in fatty acid oxidation pathways, regulation of lipid metabolism in adipose tissue, and mitigation of insulin resistance [64, 69, 70]. Together, these mechanisms contribute to the reduction of hepatic fat content. Additionally, the significance of the gut microbiome in liver health has led to the emergence of the gut-liver axis hypothesis in the pathogenesis of NAFLD [71]. Dysbiosis of the gut microbial flora disrupts crucial processes including the production of short-chain fatty acids, choline metabolism, gut mucosal integrity, and endotoxemia. These imbalances influence the expression of lipogenic genes, blood ethanol levels, and bile acid composition, thereby playing a pivotal role in the progression of hepatic steatosis [72–78]. Pomegranate peel, on the other hand, exerts inhibitory effects on the TLR4/NF-kβ pathway and enhances its antimicrobial properties through various mechanisms [79–81]. These include promoting the growth of beneficial bifidobacteria, balancing the ratio of Firmicutes to Bacteroidetes, stimulating mucin secretion, up-regulating proteins involved in maintaining tight junctions, preserving the integrity of the gastrointestinal mucosal barrier, preventing bacterial adhesion to the gut epithelium, reducing circulating levels of bacterial lipopolysaccharides, and down-regulating the expression of the TLR4 gene [27, 82–84]. These actions collectively contribute to the attenuation of inflammatory pathways implicated in the initiation and progression of hepatic steatosis [80, 85].

Our study had limitations. Lack of complete adherence to the diet in some participants is one of the limitations of this research. However, it should be noted that this is not a weakness but rather one of the limitations of lifestyle modification studies, because according to the results of published studies, only 30% of patients participating in lifestyle modification programs for the treatment of NAFLD were able to achieve the desired weight loss at the end of the year. Also, according to our knowledge, when we had first started the study, there were no RCT on fatty liver disease. Thus, we had to calculate the pomegranate peel intervention dose from related animal study in fatty liver which was consider as both strength and limitation. The animal to human dose conversion formula suggested to use the median weight of 60 kg for participants for safety considerations. However; the mean weight of our population was unknown at the beginning of study. So, we used 1500 mg of pomegranate peel which considered as safe. However, our study had strengths. This was the first study evaluating the peel of pomegranate as a therapeutic supplement which in regular life is considered as a wastage. Thus, if proved, the supplement of PP will be inexpensive, affordable and environmental friendly. Our results along with the mentioned probable underlying mechanisms suggest a valuable insights in effects of pomegranate peel on fatty liver improvement and also metabolic syndrome risk factors. In addition we suggest to determine the precise molecular pathways, and the best effective dose of supplementation further studies are suggested.

## Conclusion

In conclusion, an 8-week supplementation of pomegranate peel along with a 500 Kcal-deficit diet in NAFLD patients led to improved metabolic syndrome risk factors including lipid profile, fasting blood sugar, systolic and diastolic blood pressure, weight, fat mass and waist circumferences as well as fatty liver status in contrast to placebo.

## Data Availability

Data will be available on request.

## Abbreviations

ACAT: Acyl-CoA Cholesterol acyl transferase
ALP: Alkaline Phosphatase
ALT: Alanine Aminotransferase
AST: Aspartate Aminotransferase
BMI: Body Mass Index
CBC-Diff: Complete Blood Count with Differential Count
CRP: C-reactive Protein
CT: Computerized Tomographic
FBS: Fasting Blood Sugar
FFM: Fat Free Mass
GSH-Px: Glutathione Peroxidase
GSSR: Gastrointestinal Symptom Severity Rating
Hba1c: Haemoglobin A1C
HDL: High Density Lipoprotein
HMG-CoA: 3-Hydroxy-3-Methylglutaryl-Coenzyme A
HOMA-IR: Homeostatic Model Assessment for Insulin Resistance
IL: Interleukin
IPAQ: International Physical Activity Questionnaire
LDL: Low Density Lipoprotein
MDA: Malondialdehyde
MET: Metabolic Equivalent Task
MRI: Magnetic Resonance Imaging
MS: Metabolic Syndrome
NAFLD: Non-Alcoholic Fatty Liver Disease
NASH: Non-Alcoholic Steatohepatitis
NF-Κβ: Nuclear Factor Kappa β
PNPLA3: Patatin-Like Phospholipase Domain Containing 3
PP: Pomegranate Peel
PPAR: Peroxisome Proliferator-Activated Receptor Gamma
RCT: Randomized Clinical Trial
SOD: Super Oxide Dismutase
TC: Total Cholesterol
TG: Triglyceride
TNF-ɑ: Tumor necrosis factor ɑ
